# Predictive Factors and Nomogram for Malignant Pulmonary Nodules (≤ 1 cm)

**DOI:** 10.1155/carj/9981353

**Published:** 2026-02-25

**Authors:** Zhenxin Cao, Ying Zhu

**Affiliations:** ^1^ Xingzhi College, Zhejiang Normal University, Jinhua, 321004, China, zjnu.edu.cn; ^2^ Department of Respiratory Medicine, Jinhua Guangfu Hospital, Jinhua, 321000, China

**Keywords:** decision curve analysis, early diagnosis, lung cancer, nomogram, predictive model, pulmonary nodules, receiver operating characteristic

## Abstract

**Introduction:**

Models for predicting malignancy in pulmonary nodules ≤ 10 mm are lacking. This study aimed to identify predictive factors and develop a risk model for such nodules.

**Methods:**

A retrospective cohort study analyzed 298 patients with pulmonary nodules ≤ 1 cm. Variables including sex, smoking, nodule position, density, enhancement, diameter, and calcification were considered. A nomogram was developed using forward stepwise selection.

**Results:**

The nomogram, incorporating the seven aforementioned variables, achieved an area under the curve of 0.79. Multivariable analysis identified partial‐solid/nonsolid density (vs. solid), larger diameter, and the absence of calcification as significant independent predictors of malignancy. At its optimal threshold, the nomogram showed 70% sensitivity, 79% specificity, and 77% accuracy. Decision curve analysis indicated a net benefit.

**Conclusions:**

Nodule density, diameter, and calcification status are key independent predictors of malignancy in nodules ≤ 1 cm. The developed nomogram, which also includes other clinical and computed tomography features, shows good predictive performance but requires external validation, especially considering its sensitivity.

## 1. Introduction

Pulmonary nodules, often detected incidentally during imaging tests, are small, roundish growths in the lung that can range in size from a few millimeters to several centimeters [1, 2]. While the majority of pulmonary nodules are benign, the timely and accurate identification of malignant potential in those ≤ 1 cm is critical, as these may represent early‐stage lung cancer amenable to curative treatment [3, 4]. Epidemiologically, lung cancer is one of the leading causes of cancer‐related mortality worldwide, with small malignant pulmonary nodules posing significant diagnostic and management challenges [5–7]. These challenges often lead to patient anxiety, repeated costly imaging, or, conversely, delayed diagnosis of cancer. The social burden of lung cancer is profound, encompassing high healthcare costs, significant morbidity and mortality, and a considerable impact on patients’ quality of life [8–10].

Previous studies have explored various factors associated with the etiology, treatment response, and prognosis of lung cancer, including genetic predispositions, environmental exposures, and clinical characteristics [11–14]. However, the specific realm of malignant pulmonary nodules ≤ 1 cm remains underexplored. Despite advances in imaging techniques and biomolecular research, the heterogeneity of nodule characteristics and the limited sensitivity of current diagnostic tools contribute to the complexity of managing patients with these findings.

Our study aims to bridge this gap by identifying independent risk factors for malignant pulmonary nodules ≤ 1 cm and developing a new prediction model. The development of such a nomogram, based on readily available clinical and radiological features, holds significant clinical application value. It can empower clinicians to make more informed decisions regarding follow‐up frequency, the necessity for further diagnostic workup (e.g., positron emission tomography–computed tomography [PET–CT] and biopsy), or referral for surgical consultation, particularly in resource‐limited settings or for patients with comorbidities where invasive procedures carry a higher risk. This effort is expected to streamline diagnosis, enhance early intervention, and potentially reduce lung cancer mortality and morbidity, thereby addressing a crucial need in pulmonary oncology for more precise management of very small nodules.

## 2. Methods

### 2.1. Study Design and Participants

This study adopted a retrospective cohort design, following the STROBE guidelines for observational studies. We collected data on patients with pulmonary nodules ≤ 1 cm in diameter who underwent surgery with confirmed pathological results at Zhejiang University First Affiliated Hospital and Jinhua Guangfu Oncology Hospital from June 1, 2012, to January 20, 2025.

A total of 298 patients meeting specific criteria were included in the study. Eligible patients underwent CT scans within 1 week of hospital admission, followed by surgical confirmation via pathological results of pulmonary nodules ≤ 1 cm. It should be emphasized that the patients included in this study were all under follow‐up observation in accordance with the Chinese expert consensus on diagnosis and treatment of pulmonary nodules [2, 15–17] at that time. Specifically, every pulmonary micronodule was closely monitored, and the vast majority of patients only underwent surgical treatment after several months of follow‐up when their nodules showed a progressive trend. Therefore, this study does not involve cases where very small pulmonary nodules were surgically resected solely based on the initial diagnosis to rule out malignancy. Exclusion criteria encompassed patients with any tumor diagnosis within the past 5 years to mitigate interference from pulmonary metastatic tumors.

### 2.2. Procedure

CT scans were performed using a General Electric LightSpeed 64‐slice spiral CT scanner. Patients were positioned supine with arms raised for scans covering from the lung apex to the base, including the supraclavicular areas and axillae, using scan parameters of 120 kV and 250 mA, with a pitch of 0.984 mm and slice thickness and interval of 0.625 mm. The nodules were confirmed by chest CT, and their average diameter was calculated by averaging the largest and smallest diameters.

Image and data analysis (including assessment of nodule location, morphology, internal structure, density, enhancement, and other radiological predictor variables used in the model) were independently conducted by two senior radiologists, focusing on the nodules’ location, morphology, internal structure, and changes in the surrounding areas. In cases of disagreement, a consensus was reached through discussion.

### 2.3. Establishment of the Risk Model

In the statistical modeling phase of our study, we employed a forward stepwise selection method to identify potential predictors of malignancy in pulmonary nodules ≤ 1 cm. This approach started with a full model that included all potential predictors collected during the initial data analysis phase. Variables considered included demographic data (age and sex), smoking history, nodule characteristics (size, location, morphology, and internal structure), and radiological features (density and edge characteristics). Each step of the forward stepwise process involved evaluating the statistical significance of each variable’s contribution to the model based on the likelihood ratio test, with a predetermined significance level for entry set at *p* < 0.05. Variables not meeting this criterion were systematically removed from the model, one at a time, until only those with a significant contribution to predicting nodule malignancy remained.

Following the refinement of our predictive model via forward stepwise selection, we aimed to create a practical clinical tool: a nomogram. This graphical representation simplifies predicting clinical events, like nodule malignancy likelihood, by aligning predictor variables’ points based on regression coefficients. Utilizing the final variables from multivariable logistic regression, each predictor’s weight was scaled to assign points, generating a total score for each patient. This score determined malignancy probability, offering clinicians a user‐friendly risk estimation tool based on nodule characteristics.

Nomogram construction involved scaling predictor variables, point assignment, and creating a total point scale corresponding to malignancy probability. Internal validation techniques, like bootstrapping, assessed nomogram performance, quantified by the concordance index (C‐index). This index measures the nomogram’s ability to discriminate between patients with and without nodule malignancy, with values ranging from 0.5 (chance) to 1.0 (perfect discrimination).

### 2.4. Statistical Analysis

Analysis was carried out using SPSS Version 26.0 (IBM Corp., Armonk, NY, USA). Quantitative data were reported as mean ± standard deviation (SD), and group comparisons were analyzed using analysis of variance (ANOVA). Categorical data were expressed as numbers and percentages. Pearson correlations were used to analyze the relationships between variables. Univariable and multivariable logistic regression analyses were performed to identify significant predictors of malignancy. The predictive performance of the model was evaluated using receiver operating characteristic (ROC) curves and the area under the curve (AUC). A calibration curve and a decision curve analysis (DCA) were also performed. A forward stepwise selection method was employed for model construction, including the development of a nomogram for risk prediction. All tests were two‐sided, and a *p* value < 0.05 was considered statistically significant.

## 3. Results

### 3.1. Demographic and Clinical Characteristics

The study included 298 patients with pulmonary nodules ≤ 1 cm, with an average age of 56.19 (SD: 11.81) years. The cohort comprised 194 females (65.10%) and 104 males (34.90%). A history of previous tumors longer than 5 years was reported in 23 (7.72%) patients, whereas 275 (92.28%) had no such history. Smoking history was present in 55 (18.46%) of the participants, while 243 (81.54%) were nonsmokers. Detailed information is listed in Table 1. The benign lesions included inflammatory nodules (*n* = 22; 36.7%), hamartoma (*n* = 15, 25.0%), fibrosis (*n* = 8, 13.3%), tuberculoma (*n* = 6, 10.0%), inflammatory pseudotumor (*n* = 4, 6.7%), sclerosing pneumocytoma (*n* = 3, 5.0%), and bronchial cyst (*n* = 2, 3.3%). The malignant lesions included invasive adenocarcinoma (*n* = 145, 60.9%), adenocarcinoma in situ (*n* = 38, 16.0%), minimally invasive adenocarcinoma (*n* = 25, 10.5%), squamous cell carcinoma (SCC; *n* = 16, 6.7%), small cell carcinoma (*n* = 8, 3.4%), large cell carcinoma (*n* = 4, 1.7%), and adenosquamous carcinoma (*n* = 2, 0.8%) (Supporting Table S1). Representative pathological images are shown in Supporting Figure 1.

**TABLE 1:** Demographic characteristics.

	**Total (*n* = 298)**	**Nontumor (*n* = 60)**	**Tumor (*n* = 238)**	**p**

Age (years), mean ± SD	56.19 ± 11.81	55.67 ± 12.13	56.33 ± 11.75	0.699
Sex, *n* (%)				< 0.001
Female	194 (65.10)	28 (46.67)	166 (69.75)	
Male	104 (34.90)	32 (53.33)	72 (30.25)	
History of cancer, *n* (%)				1.000
No	275 (92.28)	55 (91.67)	220 (92.44)	
Yes	23 (7.72)	5 (8.33)	18 (7.56)	
Smoking, *n* (%)				0.010
No	243 (81.54)	42 (70.00)	201 (84.45)	
Yes	55 (18.46)	18 (30.00)	37 (15.55)	

Abbreviation: SD, standard deviation.

### 3.2. Clinical Features

As listed in Table 2, among the 298 patients, 238 had tumors, and 60 were nontumorous. Nodule location was categorized as upper (63.45% in tumor vs. 50.00% in nontumor), lower (31.51% vs. 38.33%), and right middle (5.04% vs. 11.67%), with no significant difference in distribution (*p* = 0.067). The incidence of solitary nodules did not significantly differ between tumor (50.42%) and nontumor (58.33%) groups (*p* = 0.273). No significant differences were observed in nodule smoothness, shape, or enhancement after contrast. However, differences in nodule density were statistically significant (Supporting Figure 2), with solid nodules more common in nontumors (53.33%) compared to tumors (21.01%, *p* < 0.001). Nodule diameters < 5 mm were more prevalent in the nontumor group (11.67% vs. 0.84%, *p* < 0.001), and the absence of calcification was more frequent in tumors (97.90% vs. 90.00%, *p* = 0.012). The malignancy rates were analyzed by smoking history and sex (Supporting Table S2). Among patients with malignant lesions, the female sex was associated with the nonsmoker status (*χ* ^2^ = 53.644, *p* < 0.001).

**TABLE 2:** Clinical characteristics.

	**Nontumor (*n* = 60)**	**Tumor (*n* = 238)**	**p**

Location			
Upper	30 (50.00)	151 (63.45)	0.067
Lower	23 (38.33)	75 (31.51)	
Middle‐right	7 (11.67)	12 (5.04)	
Single/multiple			
Single	35 (58.33)	120 (50.42)	0.273
Multiple	25 (41.67)	118 (49.58)	
Smoothness of boundary			
Not smooth	33 (55.00)	148 (62.18)	0.308
Smooth	27 (45.00)	90 (37.82)	
Shape			
Nonspherical	29 (48.33)	103 (43.28)	0.481
Spherical	31 (51.67)	135 (56.72)	
Density			
Solid	32 (53.33)	50 (21.01)	< 0.001
Part‐solid	20 (33.33)	118 (49.58)	
Nonsolid	8 (13.33)	70 (29.41)	
Enhancement			
Not performed	39 (65.00)	177 (74.37)	0.068
Not enhanced	11 (18.33)	44 (18.49)	
Enhanced	10 (16.67)	17 (7.14)	
Diameter			
< 5 mm	7 (11.67)	2 (0.84)	< 0.001
5–8 mm	9 (15.00)	59 (24.79)	
8–10 mm	44 (73.33)	177 (74.37)	
Calcification			
None	54 (90.00)	233 (97.90)	0.012
Yes	6 (10.00)	5 (2.10)	

### 3.3. Diagnostic Factors

In Table 3, serum tumor marker analysis showed no significant differences between tumor and nontumor groups for lung tumor markers (30.67% vs. 30.00%, *p* = 0.920), SCC antigen (2.52% vs. 5.00%, *p* = 0.561), and carcinoembryonic antigen (CEA) (3.36% vs. 3.33%, *p* = 1). The univariable analysis identified several factors with potential influence on nodule malignancy, including sex, smoking, nodule position (specifically, middle right vs. upper), nodule density (partial‐solid and nonsolid vs. solid), nodule enhancement (enhanced vs. not performed/not enhanced), nodule diameter (5–8 and 8–10 vs. < 5 mm), and calcification (presence vs. absence) in predicting malignant pulmonary nodules ≤ 1 cm (Table 4). Multivariable logistic regression analysis confirmed that nodule density with partial‐solid (odds ratio [OR] = 3.73, 95% confidence interval [CI]: 1.81–7.70, *p* < 0.001) and nonsolid (OR = 5.24, 95% CI: 1.91–14.36, *p* = 0.001) (when compared to solid density as the reference), diameters of 5–8 mm (OR = 41.36, 95% CI: 6.45–265.36, *p* < 0.001) (when compared to < 5 mm as the reference) and 8–10 mm (OR = 23.06, 95% CI: 4.04–131.70, *p* < 0.001), and calcification (OR = 0.23, 95% CI: 0.06–0.93, *p* = 0.039) (when compared to the absence of calcification as the reference) were significant independent predictors of malignancy. These findings suggest that partial‐solid or nonsolid density and larger diameters are strongly associated with the likelihood of nodule malignancy, while the presence of calcification is associated with a lower likelihood of malignancy.

**TABLE 3:** Laboratory index evaluation.

Variable	Nontumor (*n* = 60)	Tumor (*n* = 238)	*p*
Lung tumor marker panel			
Negative	42 (70.00)	165 (69.33)	0.920
Positive	18 (30.00)	73 (30.67)	
SCC			
Negative	57 (95.00)	232 (97.48)	0.561
Positive	3 (5.00)	6 (2.52)	
CEA			
Negative	58 (96.67)	230 (96.64)	1.000
Positive	2 (3.33)	8 (3.36)	

*Note:* SCC: squamous cell carcinoma antigen; CEA: carcinoembryonic antigen. Lung tumor marker panel indicates a positive result if any of the tested markers (CEA, SCC, CYFRA21‐1, and NSE) exceeded the normal reference range.

**TABLE 4:** Univariable and multivariable analyses.

Variables	Univariable analysis	*p*	Multivariable analysis	
	OR (95% CI)		OR (95% CI)	*p*
Age	1.00 (0.98–1.03)	0.698		
Sex	0.38 (0.21–0.68)	**0.001**	0.50 (0.23–1.11)	0.087
History of tumor	0.90 (0.32–2.53)	0.842		
Smoking history	0.43 (0.22–0.83)	**0.011**	0.89 (0.35–2.23)	0.800
SCC	0.49 (0.12–2.02)	0.325		
CEA	1.01 (0.21–4.88)	0.991		
Location				
Lower	0.65 (0.35–1.19)	0.163	0.84 (0.42–1.70)	0.635
Middle right	0.34 (0.12–0.94)	**0.037**	0.35 (0.11–1.09)	0.069
Single/multiple	1.38 (0.78–2.44)	0.274		
Smoothness of margin	0.74 (0.42–1.32)	0.309		
Shape	1.23 (0.70–2.16)	0.481		
Density				
Partial‐solid	3.78 (1.97–7.23)	< 0.001	3.73 (1.81–7.70)	< 0.001
Nonsolid	5.60 (2.38–13.17)	< 0.001	5.24 (1.91–14.36)	0.001
Enhancement				
Not enhanced	0.88 (0.42–1.86)	0.740	0.97 (0.41–2.28)	0.937
Enhanced	0.37 (0.16–0.88)	0.024	0.55 (0.21–1.46)	0.231
Diameter				
5–8 mm	22.94 (4.10–128.25)	< 0.001	41.36 (6.45–265.36)	< 0.001
8–10 mm	14.08 (2.83–70.14)	0.001	23.06 (4.04–131.70)	< 0.001
Calcification	0.19 (0.06–0.66)	0.008	0.23 (0.06–0.93)	0.039
Lung tumor marker Panel	1.03 (0.56–1.91)	0.920		

*Note:* SCC: squamous cell carcinoma antigen; CEA: carcinoembryonic antigen. Compared with the control group, the difference in indicators of a certain group is statistically significant (e.g., *p* < 0.05, etc.), and the significant results are highlighted in bold.

Abbreviations: CI, confidence interval; OR, odds ratio; SD, standard deviation.

### 3.4. Establishment of the Risk Model

**FIGURE 1:**
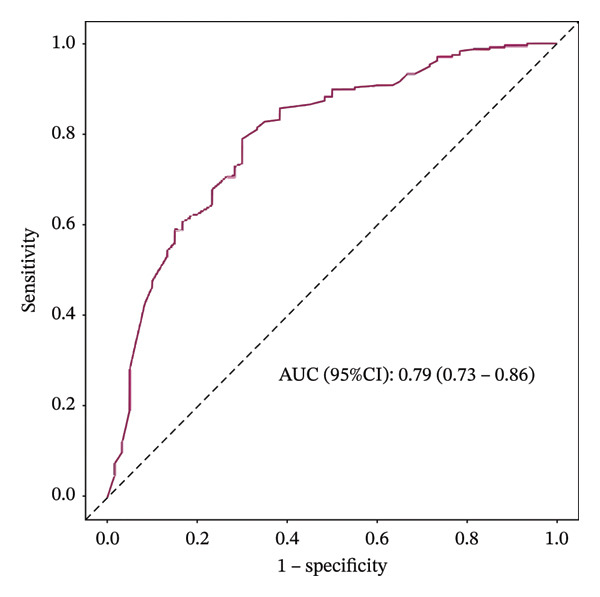
Receiver operating characteristics (ROC) curve and area under the curve (AUC) of the model for the determination of the malignancy status of pulmonary nodules ≤ 1 cm. CI: confidence interval.

**FIGURE 2:**
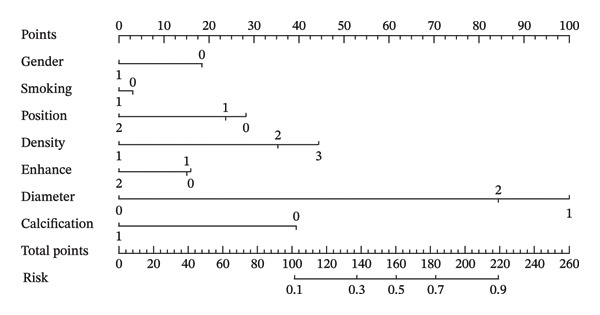
Nomogram construction for the determination of the malignancy status of pulmonary nodules ≤ 1 cm. The nomogram predicts the probability of malignancy based on seven risk factors. To use it, locate the patient’s value on each variable axis, draw a vertical line upward to the “Points” axis to determine the score, sum the scores for all variables, and locate the total score on the “Total Points” axis to find the corresponding risk probability. Variable definitions: gender: 0 = female, 1 = male; smoking: 0 = No, 1 = Yes; position: 0 = ppper lobe, 1 = lower lobe, 2 = middle right lobe; density: 1 = solid, 2 = part‐solid, 3 = nonsolid; enhancement: 0 = not performed, 1 = not enhanced, 2 = enhanced; diameter: 0 = < 5 mm, 1 = 5–8 mm, 2 = 8–10 mm; calcification: 0 = absent, 1 = present.

In this study, the forward selection method was used to screen variables. Ultimately, seven variables were included in the final analysis: sex, smoking, nodule position, nodule density, nodule enhancement, nodule diameter, and calcification. The selected variables were used to develop a nomogram model to predict the malignancy of pulmonary nodules ≤ 1 cm. When combined into the nomogram model, the seven parameters achieved an AUC of 0.79, suggesting a good predictive performance, as AUC values above 0.7 are generally considered indicative of a useful predictive tool (Figure 1). The nomogram is shown in Figure 2.

The optimal threshold for the nomogram model was identified as 0.755, which was selected to maximize the balance between sensitivity and specificity. The AUC was 0.79 (95% CI: 0.73–0.86). At this threshold, the model demonstrated a sensitivity of 70%, a specificity of 79%, and an overall accuracy of 77%. The positive predictive value (PPV) and negative predictive value (NPV) were 46% and 91%, respectively (Table 5). The calibration curve showed that the model line generally followed the ideal curve (Figure 3(a)). The DCA also shows a tradeoff of the model curve between the treat‐none and treat‐all curves (Figure 3(b)). These metrics underscore the model’s ability to accurately identify malignant nodules, with a sensitivity of 70% indicating that a proportion of malignant nodules might be missed.

**TABLE 5:** The performance of the model.

AUC (95% CI)	Accuracy (95% CI)	Sensitivity (95% CI)	Specificity (95% CI)	PPV (95% CI)	NPV (95% CI)	Cut off
0.79 (0.73–0.86)	0.77 (0.72–0.82)	0.70 (0.58–0.82)	0.79 (0.74–0.84)	0.46 (0.35–0.56)	0.91 (0.87–0.95)	0.755

*Note:* AUC: area under the curve.

Abbreviations: CI, confidence interval; NPV, negative predictive value; PPV, positive predictive value.

FIGURE 3Evaluation of the nomogram model. (a) Calibration curve and (b) decision curve analysis (DCA) for the determination of the malignancy status of pulmonary nodules ≤ 1 cm.

**Figure:**
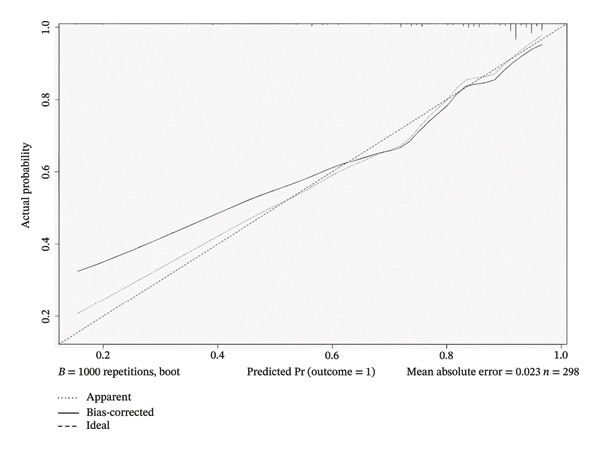
(a)

**Figure:**
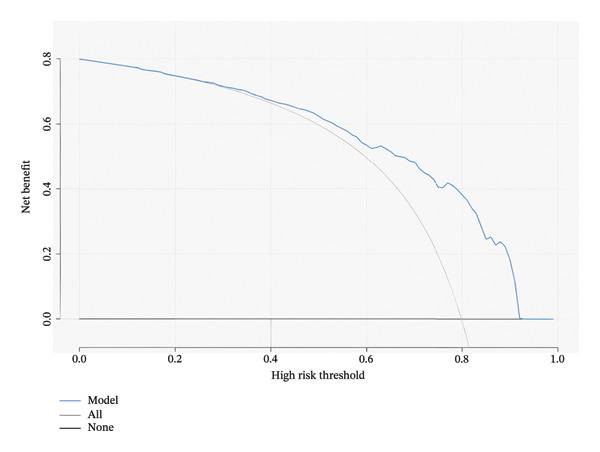
(b)

## 4. Discussion

In our investigation into the predictive factors for the progression of malignant micronodules to lung cancer, a nomogram for predicting the progression of malignant micronodules in early diagnosis was developed using factors such as sex, smoking, nodule position, nodule density, nodule enhancement, nodule diameter, and calcification. This delineation of predictors is particularly crucial in the context of precision medicine, where the ability to accurately forecast nodule malignancy can significantly enhance early diagnosis and treatment strategies. Our findings suggest that these seven variables play a pivotal role in determining the likelihood of a micronodule developing into lung cancer, emphasizing the value of detailed nodule characterization in clinical practice.

In the era of precision medicine, the statistical analysis of the malignancy rate and pathological type characteristics of malignant pulmonary micronodules represents a pivotal step toward effective early warning, diagnosis, and clinical treatment strategies, ultimately aiming to extend the survival of lung cancer patients. The exploration of differences in clinical and radiological characteristics among patients with pulmonary nodules of various pathological types is crucial for guiding clinical practice toward better judgment of the nature and pathological types of lung nodules.

Although models and biomarkers (such as CEA) exist for predicting malignancy in incidental pulmonary nodules ≤ 20 mm, there remains a marked lack of predictive models specifically for nodules ≤ 10 mm [18–21]. Cui et al. [22] addressed this gap by developing a model utilizing four CT parameters (tumor–lung interface, spiculation, air bronchogram, and invisibility at the mediastinal window), achieving an AUC of 0.781–0.875. Similarly, two deep learning studies leveraging CT data reported AUCs ranging from 0.754–0.942 [23] and 0.645–0.964 [24] for nodules < 1 cm, while another CT/radiomics model provided AUCs of 0.930–0.942 [25]. Nonetheless, deep learning models have limitations for routine use because they require specialized software, depend on training cohort characteristics, and largely function as a “black box” [26]. In contrast, a nomogram derived from easily accessible clinical data offers better clinical applicability. This highlights the innovation and value of our study, which aims to set a new benchmark in precision predictive modeling for pulmonary micronodules. Through regression analyses of clinical data from pathologically confirmed micronodule cases, our research identifies independent predictors of malignancy and evaluates the model’s diagnostic performance against the classic Mayo Clinic Model [16, 27]. Additionally, the development of this nomogram, which integrates clinical and radiological features, is designed to offer a precise diagnostic framework. By aggregating accessible clinical data, this tool enables refined risk stratification and supports informed clinical decision‐making. Beyond established models like the Mayo Clinic and Brock models [28, 29], our retrospective incorporation of part‐solid nodules furthers the identification of independent malignancy predictors in micronodules and facilitates a novel diagnostic approach. The resulting MAP‐based decision support system provides rapid clinical guidance for characterizing pulmonary nodules and optimizing management, helping minimize patient anxiety and overtreatment.

Our retrospective study identified independent predictors for small pulmonary nodules and proposed a new diagnostic model. The MAP‐based decision support system marks significant progress in pulmonary nodule management, enabling rapid decisions on nodule characterization and therapy while minimizing patient stress and overtreatment. By integrating imaging and clinical data, this approach demonstrates the value of precision medicine, highlighting how advanced analytics can elevate diagnostic accuracy and outcomes. Importantly, our findings contrast with earlier studies on tumor markers SCC and CEA [30, 31], as these markers did not act as discriminative predictors in our cohort. This discrepancy may result from elevated SCC and CEA levels found in lung infections within nontumor samples, suggesting that inflammation potentially confounds biomarker‐based distinctions. These insights argue for caution when relying on such markers in nodule assessment, particularly for patients presenting with concurrent infectious conditions.

A noteworthy observation in this study is the greater proportion of malignant nodules found in nonsmokers compared to smokers. While this result may seem counterintuitive, it is actually consistent with epidemiological patterns reported in East Asian populations, especially regarding lung adenocarcinoma. In fact, numerous studies have documented that the incidence of lung adenocarcinoma among nonsmoking women in East Asia substantially exceeds that seen elsewhere [32–36]. The present study also showed that the proportion of nonsmokers was higher in women than in men. Given that women comprised 65.1% of the study cohort and most were nonsmokers, this demographic characteristic could help clarify the findings. These results underscore the importance of maintaining a high degree of clinical vigilance for pulmonary nodules in nonsmokers, with particular attention to female patients.

Multivariable analysis revealed that the OR for malignancy was higher in nonsolid nodules than in part‐solid nodules. This finding is consistent with the pathological nature of early‐stage adenocarcinomas, as many present as pure ground‐glass opacities, which are characteristic of adenocarcinoma in situ or minimally invasive adenocarcinoma. A second, counterintuitive finding was that the OR for the 5–8‐mm size group surpassed that of the 8–10‐mm group. We identified this as a statistical artifact stemming from the instability caused by the very small reference group (< 5 mm, *n* = 9), which leads to unreliable OR estimates and overly wide CIs. To confirm this, we performed a sensitivity analysis by changing the reference group to the more populous “5–8‐mm” category. This analysis confirmed that the < 5‐mm group had a significantly lower risk of malignancy (OR = 0.06; 95% CI: 0.01–0.31), while no significant difference was found between the 8–10 mm group and the 5–8‐mm group (OR = 0.68; 95% CI: 0.27–1.57). This confirms that the original finding was driven by statistical instability. Therefore, the most robust conclusion is not to focus on a nonlinear risk relationship but on the overall trend: nodules measuring ≥ 5 mm are considerably more likely to be malignant than those < 5 mm. Future studies with larger cohorts are needed to better define the precise risk gradient.

Regarding clinical application, the optimal cutoff value determined by the Youden index in our model was 0.755. Correlating this with the nomogram, a total score of approximately 190 points serves as the recommended threshold for identifying patients who may benefit from active clinical intervention. Furthermore, scores exceeding 220 points indicate a probability of malignancy approaching 90%, distinguishing a subset of patients at significantly elevated risk that warrants immediate attention.

The study has several limitations that warrant consideration. First, the retrospective design and a relatively small sample size may limit the statistical power and robustness of our findings. A crucial next step is external validation in a larger, independent cohort to confirm the model’s generalizability. Second, the study is susceptible to significant selection bias. Our cohort was exclusively composed of patients who underwent surgical resection following documented nodule progression, such as interval growth or the development of a solid component. This inclusion criterion inherently enriched the study population with high‐risk lesions, meaning the model’s reported predictive performance is likely overestimated when applied to a general population of incidental small nodules. Finally, our analysis was restricted to variables available in existing medical records, which may have omitted other potential predictive factors.

Looking forward, the insights gleaned from this study underscore the complex interplay of factors influencing the progression of pulmonary micronodules to cancer. They highlight the imperative for larger, prospective studies to validate and refine predictive models, incorporating a broader spectrum of variables, including but not limited to, genetic markers, patient lifestyle, and environmental exposures. Such comprehensive approaches are crucial for advancing our understanding and enhancing the accuracy of lung cancer prediction models, ultimately contributing to improved patient outcomes through early detection and personalized treatment strategies.

## 5. Conclusion

Sex, smoking, nodule position, nodule density, nodule enhancement, nodule diameter, and calcification were used to develop a nomogram for malignant micronodule progression in early diagnostic prediction. While the developed nomogram showed promising predictive performance (AUC: 0.79), limitations in the study design and suboptimal DCA outcomes warrant caution in clinical application. Discrepancies with prior literature on tumor markers like SCC and CEA suggest potential confounding effects of nontumor conditions like inflammation. Further research, particularly larger, prospective studies with comprehensive validation cohorts, is crucial to refine predictive models for pulmonary nodules. Advancements in pulmonary oncology hinge on accurate early detection and tailored treatment strategies, aiming to improve patient prognosis and lessen the disease burden.

## Author Contributions

Ying Zhu carried out the studies, participated in collecting data, and drafted the manuscript. Zhenxin Cao performed the statistical analysis and participated in its design. Ying Zhu and Zhenxin Cao participated in the acquisition, analysis, or interpretation of data and drafted the manuscript.

## Funding

This work was supported by the Public Welfare Project of the Jinhua Science and Technology Bureau (Project Number: 2020‐4–077) and the Zhejiang Provincial Science and Technology Plan Project of China (No. 2023C35089).

## Disclosure

All authors read and approved the final manuscript.

## Conflicts of Interest

The authors declare no conflicts of interest.

## Supporting Information

Supporting Figure 1: Representative pathological results of tumor (left) and nontumor (right).

Supporting Figure 2: Representative CT results of tumor (left) and nontumor (right).

Supporting Table S1: Diagnoses of the included lesions.

Supporting Table S2: Counts of malignant cases by sex and smoking status (*n* = 238).

## Supporting information


**Supporting Information** Additional supporting information can be found online in the Supporting Information section.

## Data Availability

All data generated or analyzed during this study are included in this published article.
