# Supplementary material for: Predictive Factors and Nomogram for Malignant Pulmonary Nodules (≤ 1 cm)
Source: Can Respir J. 2026 Feb 25;2026:9981353. doi: 10.1155/carj/9981353 (PMC12933631; doi:10.1155/carj/9981353)
Supplement: Supplementary file 1 — Supporting Information Additional supporting information can be found online in the Supporting Information section. [file CARJ-2026-9981353-s001.docx]

**Supplementary Figure 1** Representative pathological results of tumor (Left) and non-tumor (Right).

10X


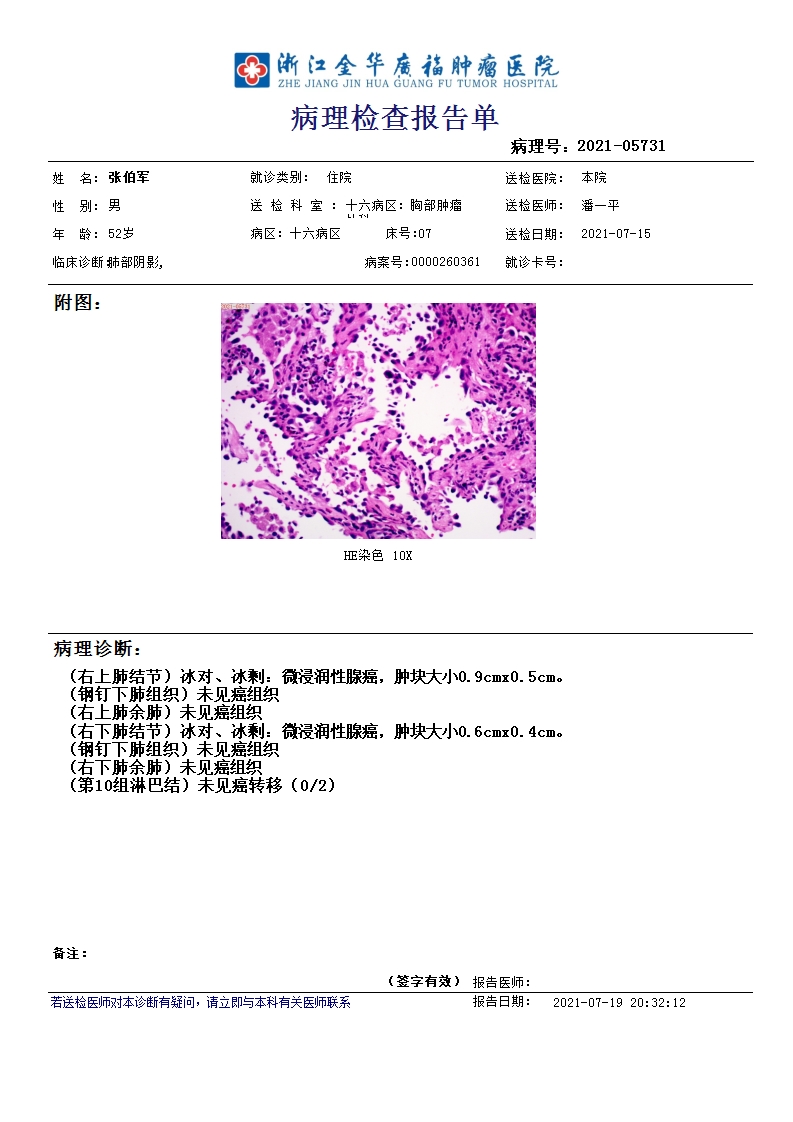

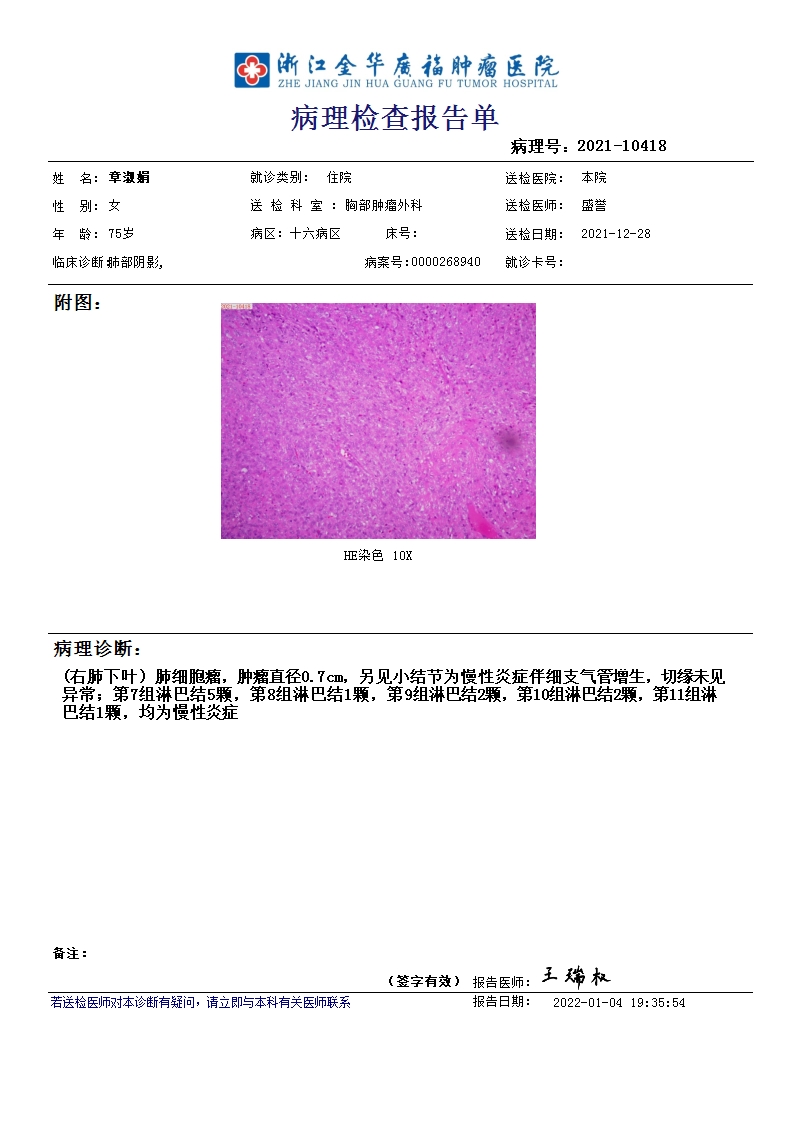


**Supplementary Figure 2** Representative CT results of tumor (Left) and non-tumor (Right).


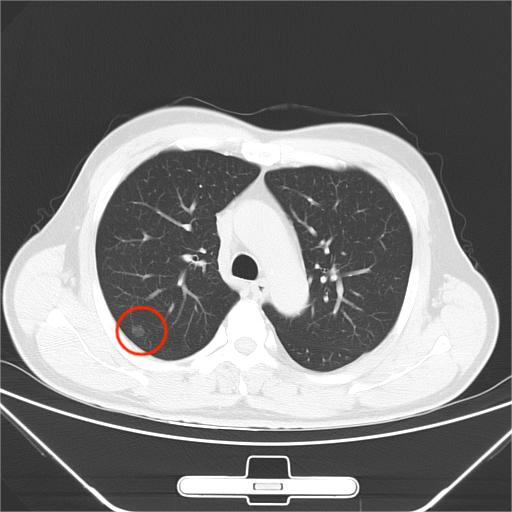

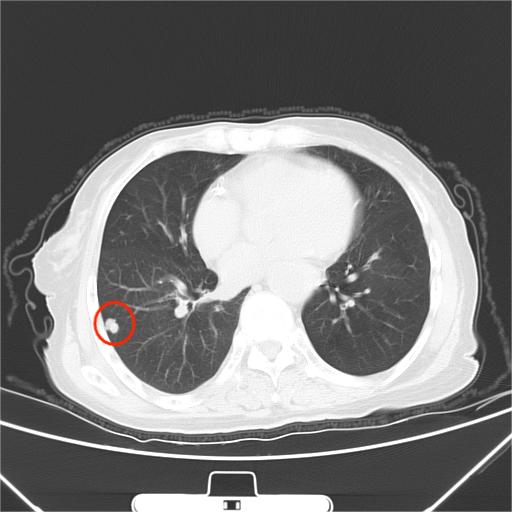


**Supplementary Table S1.** Diagnoses of the included lesions.

| **Lesion type** | **Pathological type** | **n** | **%** |
| --- | --- | --- | --- |
| Benign (n = 60) | Inflammatory nodule | 22 | 36.67 |
|  | Hamartoma | 15 | 25.00 |
|  | Fibrosis | 8 | 13.33 |
|  | Tuberculoma | 6 | 10.00 |
|  | Inflammatory pseudotumor | 4 | 6.67 |
|  | Sclerosing pneumocytoma | 3 | 5.00 |
|  | Bronchial cyst | 2 | 3.33 |
| Malignant (n = 238) | Invasive adenocarcinoma | 145 | 60.92 |
|  | Adenocarcinoma in situ | 38 | 15.97 |
|  | Minimally invasive adenocarcinoma | 25 | 10.50 |
|  | Squamous cell carcinoma | 16 | 6.72 |
|  | Small cell carcinoma | 8 | 3.36 |
|  | Large cell carcinoma | 4 | 1.68 |
|  | Adenosquamous carcinoma | 2 | 0.84 |

**Supplementary Table S2.** Counts of malignant cases by sex and smoking status (n=238)

|  | **Smoking History: Yes** | **Smoking History: No** | **Total** |
| --- | --- | --- | --- |
| Male | 30 | 42 | 72 |
| Female | 7 | 159 | 166 |
| Total Smoking | 37 | 201 | 238 |
